# An evaluation of anatomical and functional outcomes in patients undergoing retinectomy for proliferative vitreoretinopathy

**DOI:** 10.1186/s12886-026-04904-8

**Published:** 2026-05-13

**Authors:** Dilek Uzlu, Hidayet Erdöl, Ömer Berk Bulanık, Murat Günay, Büşra Köse, Mehmet Kola

**Affiliations:** Medical Faculty, Department of Ophthalmology, Karadeniz Technical University, Trabzon, Turkey

**Keywords:** Proliferative vitreoretinopathy, Retinectomy, Retinotomy, Pars plana vitrectomy, Retinal detachment

## Abstract

**Background:**

The objective of this study was to evaluate the anatomical and functional outcomes of patients who underwent relaxing retinotomy or retinectomy for retinal detachment complicated by proliferative vitreoretinopathy.

**Methods:**

The medical records of all patients who underwent retinectomy for retinal detachment complicated with proliferative vitreoretinopathy between 2015 and 2023 were reviewed retrospectively.

**Results:**

The patients’ mean age was 60.83 ± 16.4 (21–90) years. The study population consisted of 28 male (42.4%) and 38 female (57.6%). The mean follow-up period was 21.72 ± 13.44 (12–65) months. The mean initial visual acuity was 2.1 ± 0.63 logMAR, while the mean final visual acuity was 1.94 ± 0.69 logMAR (*p* = 0.094). Twenty-one patients presented with rhegmatogenous retinal detachment (31.8%), 36 with tractional retinal detachment due to diabetes (54.5%), and nine with retinal detachment secondary to trauma (13.6%). Visual acuity increased in 30 patients (45.45%), decreased in 10 patients (15.15%), and did not change in 26 patients (39.39%). Mean intraocular pressure (IOP) values were 13.25 ± 4.67 mmHg (range 5–28) at baseline and 12.98 ± 4.75 mmHg (range 4–25) at the final examination. Twenty-seven patients underwent 90-degree retinectomy (40.9%), 18 underwent 180-degree retinectomy (27.3%), 6 underwent 270-degree retinectomy (9.1%), and 15 underwent 360-degree retinectomy (22.7%). When evaluating proliferative retinal detachment by etiology (rhegmatogenous, traumatic, diabetic), no statistically significant differences were observed in baseline and final visual acuity or intraocular pressure (*p* > 0.05), nor in recurrence rates (*p* = 0.42).

**Conclusion:**

The findings of this study indicate that retinectomy is a beneficial treatment for cases of difficult retinal detachment, particularly when there is a need for both functional vision and retinal stabilization.

## Introduction

Proliferative vitreoretinopathy (PVR) results from cell migration into the vitreous, proliferation, the development of epiretinal fibrocellular membranes, and intraretinal fibrosis [1, 2]. The condition can be conceptualized as a reparative process initiated by the tear that precipitates retinal detachment. Experimental research has shown that intraretinal proliferation of the retinal pigment epithelium, Müller cells, and other non-neural cells occurs following rhegmatogenous retinal detachment [3, 4]. PVR has been documented in 5–10% of patients following rhegmatogenous retinal detachment [5, 6]. Several factors have been identified as increasing the risk of PVR, including trauma, large and giant retinal tears, and chronic retinal detachment. PVR represents the most prevalent and significant cause of failure in retinal detachment surgery [7, 8].

The development of PVR represents a significant complication, necessitating the use of intricate surgical techniques in treatment. Despite recent advancements made in vitreoretinal surgery, considerable problems persist in the management of PVR. The objective of the surgical procedure is to occlude the retinal hole, relieve traction, stabilize the retina, and reduce the likelihood of recurrence. Successful outcomes can be achieved through the implementation of various surgical techniques, including scleral buckling, pars plana vitrectomy (comprehensive cleaning of the vitreous base), membrane peeling, relaxing retinotomy and retinectomy, cryopexy or endolaser photocoagulation, and intraocular tamponade [9–11].

In cases of posterior PVR, surgical removal of the membranes is typically feasible, rendering the need for relaxing retinotomy rare. Nonetheless, the dissection of peripheral membranes in anterior PVR patients constitutes a more challenging surgery, often necessitating retinotomy. In some cases, retinal folds and rigidity develop as a result of extensive fibrosis, contraction and retinal atrophy. Such complications *result in* anteroposterior and circumferential traction of the retina (anterior PVR), especially at the vitreous base. When retinotomy is judged essential, it is crucial to execute the treatment with accuracy and diligence to entirely alleviate the traction and tension in the retina. As originally described by Machemer, retinotomies should be large, peripheral, clean, and straight in order to treat both intraretinal anteroposterior contraction and subretinal bands [10].

This study investigated anatomical and functional outcomes following relaxing retinotomy/retinectomy due to complicated retinal detachment with PVR.

## Materials and methods

The medical records of all retinectomy cases with complicated retinal detachment at the Karadeniz Technical University Faculty of Medicine, Türkiye, between 2015 and 2023 were investigated retrospectively. The study was carried out in accordance with the principles of the Declaration of Helsinki and approved by the Local Institutional Ethics Committee of University of Karadeniz Technical University, Faculty of Medicine (decision no:24237859-360, date:14.03.2025). Informed consent was obtained from all individual participants included in this study. The etiology of the retinal detachment (rhegmatogenous, tractional, or traumatic), preoperative visual acuity and intraocular pressure, the extent of the retinectomy, intraoperative tamponade, and laser treatments were documented. Cases with uveitis (such as acute retinal necrosis) and cases secondary to endophthalmitis, patients with incomplete file data and less than one year of follow-up after PPV were excluded. Of the 76 patients who underwent retinectomy between 2015 and 2023, 66 were included in the study. All patients who underwent retinectomy had anterior PVR. Posterior membranes were peeled away as much as possible using forceps. Patients who experienced a recurring detachment beneath the silicone oil or a detachment subsequent to silicone oil removal were classified as having relapsed. Postoperative improvement in the retina and macula, final intraocular pressure (IOP), and final visual acuity were evaluated.

All retinal surgeries were performed by the same author (H.E.). In all cases, the pre-retinal membranes were peeled toward the anterior as far as possible after vitreous clearance using a 23-gauge three-port pars plana technique. The retinectomy areas were delineated with diathermy to facilitate hemostasis. A vitrectomy probe was used to perform the retinectomy, with the objective of achieving optimal ablation of the anterior retinal flap. Perfluorocarbon liquids or fluid-air exchange were applied to the retina. An endolaser was then applied to the retinectomy margin. All cases underwent 360-degree retinal laser photocoagulation. A silicone oil tamponade (1000 cSt) was employed to all patients. Simultaneous phacoemulsification surgery was performed on patients with cataracts.

### Statistical analysis

Statistical analysis was conducted using SPSS version 21 software (IBM Corp., Armonk, NY, USA). The Shapiro-Wilk test was applied to assess the suitability of the normal distribution. Nonparametric tests were performed for data that did not conform to a normal distribution. The Kruskal-Wallis test was used for comparing multiple groups. For comparisons involving three groups, pairwise comparisons with Bonferroni correction were performed for variables found to be significant after the global test. The Mann-Whitney U test was employed for mean comparisons when the data were not normally distributed, while the t-test was applied for parametric analyses. The chi-square test was employed for comparing proportions. Fisher’s chi-square test was performed when the number of variables per cell was less than 5. Pearson’s test was applied for parametric correlation analysis, and Spearman’s test for non-parametric correlation analysis. Logistic regression analysis was also performed to determine the effect of variables (age, initial visual acuity, initial lens status, retinectomy extent, etiology, and number of previous surgeries) on final vision. A p-value less than 0.05 was considered to indicate statistical significance.

## Results

Sixty-six patients who underwent retinectomy between 2015 and 2023 were finally enrolled in the study. The mean age of these 66 patients was 60.83 ± 16.4 years (range: 21–90 years), 28 (42.4%) were male, and 38 (57.6%) were female. In all patients, the evaluation was conducted on a single eye. The mean follow-up duration was 21.72 ± 13.44 months (range: 12–65 months). Twenty patients had no previous history of PPV surgery, 30 patients had undergone one previous PPV procedure, 15 patients two procedures, and one patient had undergone three previous PPV surgeries. The mean number of previous vitrectomy surgeries was 0.71 ± 0.76 (0–3). A history of trauma was present in nine patients (13.6%) and was characterized by penetrating eye injuries, which were treated by means of primary ocular repair surgery.

**Table 1:** The patients’ baseline characteristics

Age (years)	60.83 ±16.4
Gender	
Male	28(42.4%)
Female	38 (57.6%)
Initial VA (logMAR)	2.1±0.63
Initial IOP (mmHg)	13.25±4.67
Number of previous surgeries (PPV)	
0	20
1	30
2	15
3	1
Lens condition	
Phakic	29 (43.9%)
Pseudophakic	27 (40.9%)
Aphakic	10 (15.2%)
Retinectomy Degree	
90	27
180	18
270	6
360	15

Mean baseline intraocular pressure (IOP) was 13.25 ± 4.67 mmHg (range 5–28 mmHg), while the mean IOP at the final examination was 12.98 ± 4.75 mmHg (range 4–25 mmHg). Twenty-nine (43.9%) patients were phakic, 27 (40.9%) were pseudophakic, and 10 (15.2%) were aphakic. Fifteen patients underwent simultaneous cataract and pars plana vitrectomy surgery due to cataract. Twenty-seven patients underwent 90-degree retinectomy during the surgical procedure (40.9%), 18 underwent 180-degree retinectomy (27.3%), six underwent 270-degree retinectomy (9.1%), and 15 underwent 360-degree retinectomy (22.7%) (Table 1). The retinectomies were typically conducted in a location anterior to the equator. Mean baseline visual acuity was 2.1 ± 0.63 logMAR, and mean final visual acuity was 1.94 ± 0.69 logMAR (*p* = 0.094). Thirty patients (45.45%) exhibited an improvement in visual acuity compared to baseline, 10 patients (15.15%) a decline, and 26 patients (39.39%) no change.

Twenty-one patients presented with rhegmatogenous retinal detachment (31.8%), 36 with tractional retinal detachment due to diabetes (54.5%), and nine with retinal detachment secondary to trauma (13.6%). PVR grading in patients with rhegmatogenous retinal detachment and traumatic retinal detachment was classified in accordance with the system proposed by the Retina Society Terminology Committee (1983) [12]. PVR was grade C3 in 14 patients and grade D in 16 patients (D1 11, D2 2 and D3 3 patients).

The mean ages were 63.05 ± 17.31 years in rhegmatogenous retinal detachment group, 61.36 ± 16.29 in diabetes tractional retinal detachment group, and 53.56 ± 14.21 in traumatic retinal detachment group. No statistically significant differences were determined among the three groups (*p* = 0.33). Mean numbers of previous PPV operations were 0.71 ± 0.76 in rhegmatogenous retinal detachment group, 0.49 ± 0.65 in diabetes tractional retinal detachment group, and 1.56 ± 0.72 in traumatic retinal detachment group. The numbers varied significantly between the groups, with the traumatic retinal detachment group necessitating a greater number of surgeries (*p* = 0.001).

Mean visual acuity at baseline was 1.99 ± 0.56 logMAR in the rhegmatogenous retinal detachment group, 2.05 ± 0.66 in the diabetic tractional retinal detachment group, and 2.57 ± 0.46 logMAR in the traumatic retinal detachment group. Although the lowest visual acuity was observed in the trauma group, the intergroup differences were not statistically significant (*p* = 0.07). Final visual acuity was 2.03 ± 0.64 logMAR in the rhegmatogenous retinal detachment group, 1.83 ± 0.74 logMAR in the diabetes tractional retinal detachment group, and 2.14 ± 0.69 logMAR in the traumatic retinal detachment group. No significant differences were also determined in terms of final visual acuity between the groups (*p* = 0.45). Table 2 illustrates the intra-group changes in visual acuity and IOP.

**Table 2:** The comparisons of visual acuity and IOP

	BCVA (logMAR)		IOP(mmHg)	
**Rhegmatogenous retinal detachment group** Preoperative Postoperative	1.99 ± 0.56 2.03 ± 0.64	*P* = 0.94	12.71 ± 4.60 13.05 ± 5.71	*P* = 0.93
**Diabetic tractional retinal detachment group** Preoperative Postoperative	2.05 ± 0.66 1.83 ± 0.74	*P* = 0.01	14.29 ± 4.43 14.34 ± 3.98	*P* = 0.96
**Traumatic retinal detachment group** Preoperative Postoperative	2.57 ± 0.46 2.14 ± 0.69	*P* = 0.04	9.78 ± 5.01 11.40 ± 3.34	*P* = 0.14

Regarding the extent of retinectomy based on etiology, less extensive retinectomies were necessary for rhegmatogenous retinal detachment, but comprehensive retinectomies up to 360 degrees were mandated in the tractional and traumatic categories (Table 3).

**Table 3:** Degrees of retinectomy according to etiology

Degree of Retinectomy	Rhegmatogenous	Tractional	Traumatic	Total
90°	13(61.9%)	12(33.3%)	2(22.2%)	27
180°	7(33.3%)	9 (25.0%)	2(22.2%)	18
270°	1(4.8%)	5 (13.9%)	0(0%)	6
360°	0(0%)	10(27.8%)	5(55.6%)	15
Total	21 (100%)	36(100%)	9(100%)	66

**Fig. 1:**
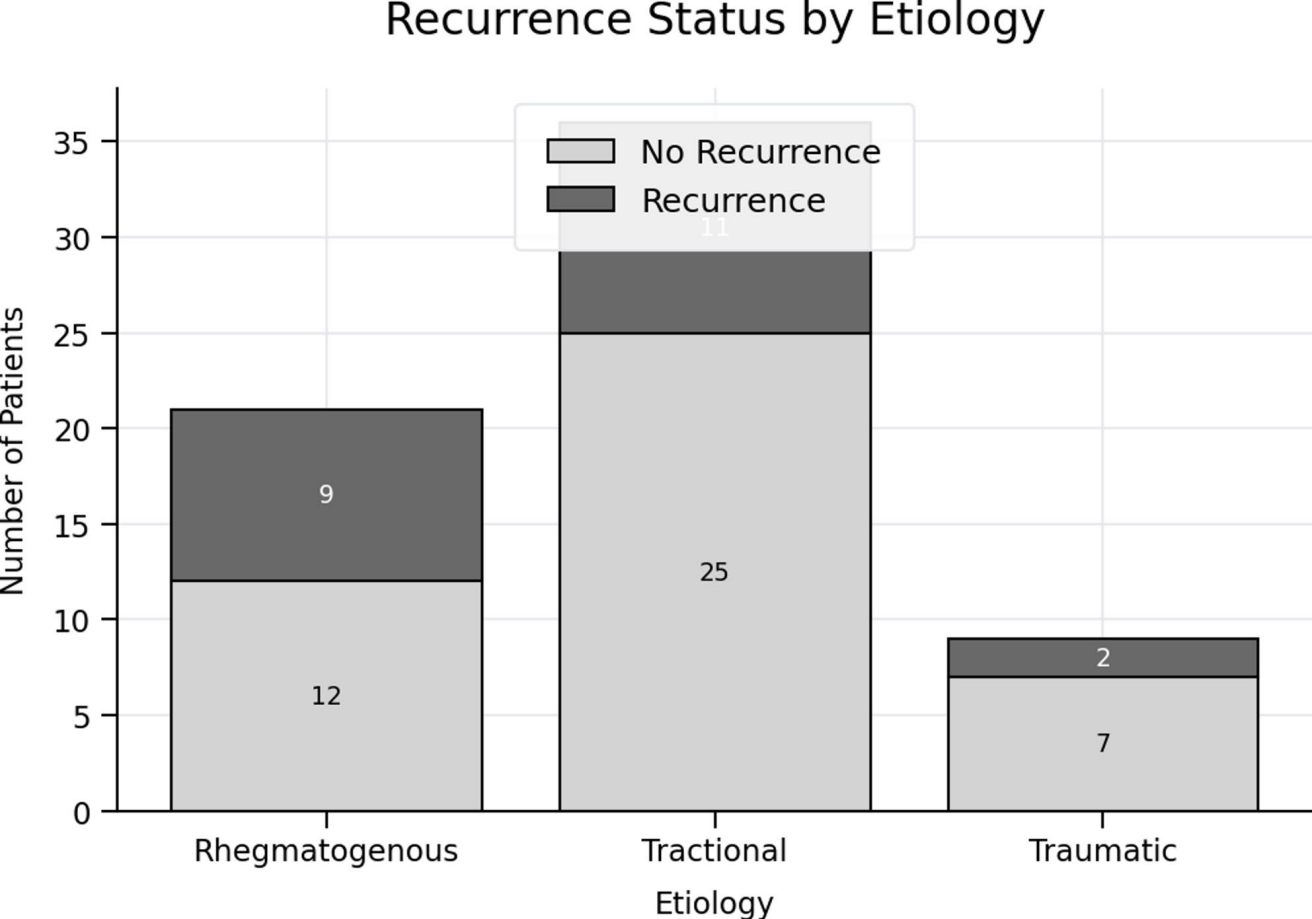
Recurrence rates by etiology

**Fig. 2:**
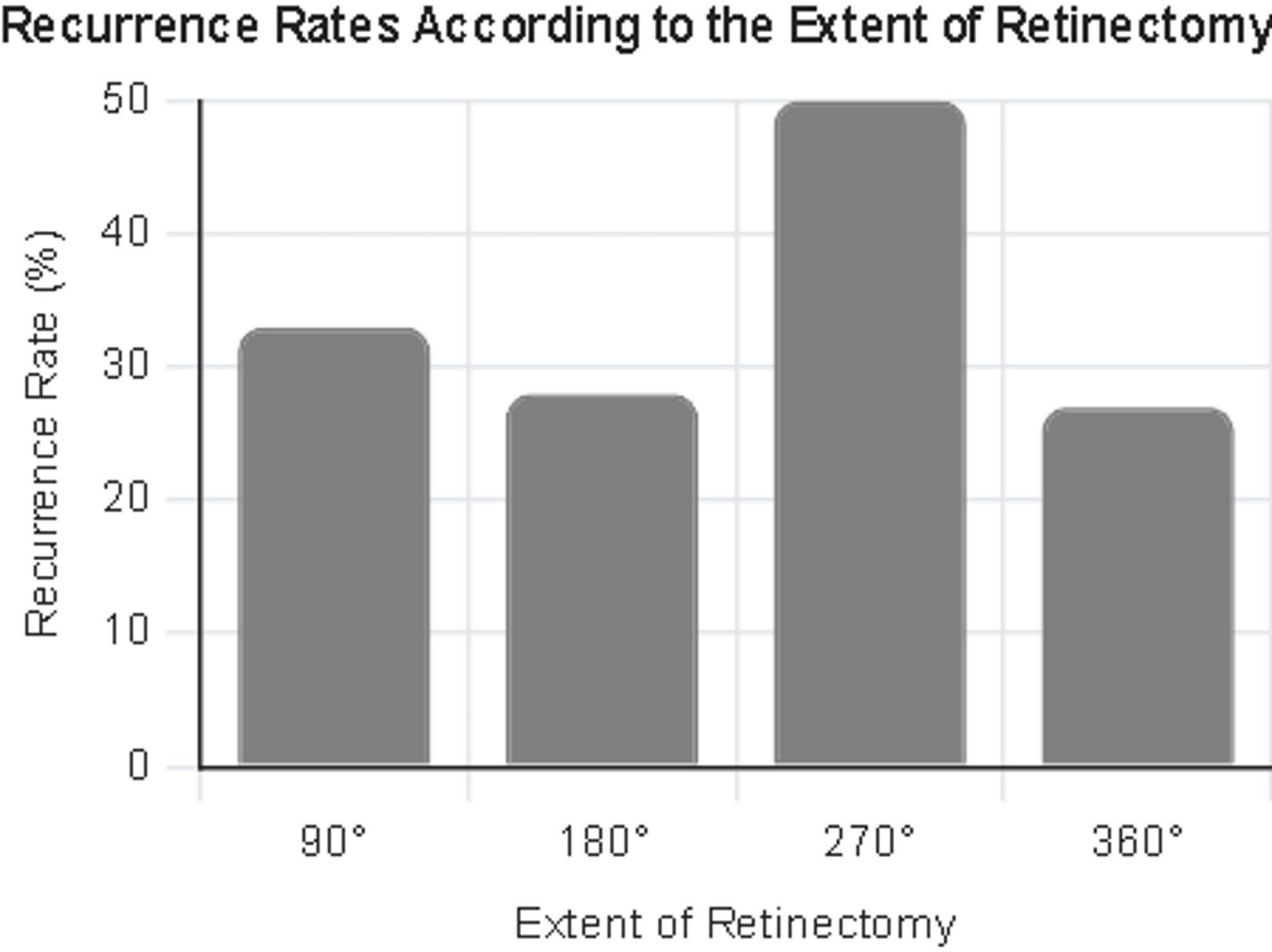
Recurrence rates according to the extent of retinectomy

During the follow-up period, 44 patients (66.7%) exhibited no recurrence, while 22 patients (33.3%) experienced recurrence. Recurrence was observed in 12 patients under silicone oil and in 10 patients following its removal. No statistically significant difference in recurrence rates was found among the three groups (*p* = 0.42) (Fig. 1). No substantial difference was observed in retinectomy size or recurrence rates (*p* = 0.60) (Fig. 2).

The mean duration for silicone removal was 5.4 ± 2.3 months. Hypotony (≤ 5 mmHg) occurred in 5 eyes (7.5%). Five patients had intraocular pressure over 25 mmHg and were initiated on a singular anti-glaucoma medication (7.5%).

A logistic regression analysis was performed to evaluate the effects of variables on final vision and recurrence. The variables included age, initial visual acuity, initial lens status, retinectomy degree, etiology, and number of previous surgeries. The analysis revealed that age, initial visual acuity level and etiology had a significant effect (Table 4).

**Table 4:** Logistic regression results for the final visual acuity

Parameters	OR	95%CI	*P* value
Age	0.937	0.883–0.994	**0.031**
Initial visual acuity	6.787	1.579–29.17	**0.010**
Initial lens status	2.328	0.623–8.703	0.209
Retinectomy degree	1.302	0.665–2.548	0.441
Etiology	0.150	0.036–0.628	**0.009**
Previous operation number	0.657	0.217–1.987	0.457

OR: Odds Ratio; Model: Pseudo R²=0.092, LLR p=0.186. CI: Confidence Interval

Each increase in age reduces the probability of poor outcome by approximately 6.3%. This finding suggests that patients who underwent surgery at a younger age (expected to be more frequent, especially in the trauma group) may show better outcomes with a longer follow-up period despite relatively poor baseline conditions. In patients with poor baseline visual acuity, the probability of poor vision in the first year increases 6.8 times.

In terms of etiology, the probability of poor vision was found to decrease progressively in the order of rhegmatogenous, traction, and trauma. Lens status, retinectomy degree, and number of previous surgeries were not statistically significant (*p* > 0.05).

When the same analysis was performed for the probability of recurrence, age was found to be insignificant at the borderline, and the other variables were not significant (Table 5).

**Table 5:** Logistic regression results for recurrence

Parameters	OR	95%CI	*P* value
Age	0.969	0.934–1.006	0.097
Initial visual acuity	0.794	0.327–1.927	0.610
Initial lens status	0.960	0.557–1.656	0.884
Retinectomy degree	0.509	0.190–1.361	0.178
Etiology	1.570	0.703–3.505	0.271

OR: Odds Ratio; Model: Pseudo R²=0.092, LLR *p* = 0.186. CI: Confidence Interval

## Discussion

Proliferative vitreoretinopathy is a cellular activity involving the proliferation and contraction of retinal pigment epithelial cells, glia cells, fibroblasts, and macrophages. The standard treatment of PVR is surgery. In advanced cases, retinotomies and retinectomies and the use of intravitreal buffers are required. Traction at the vitreous base represents the most frequent cause of failure or recurrence post-PVR surgery. The dense collagen fibrils located in the vitreous base extend vertically to the retina and pars plana and adhere tightly to the internal limiting membrane. Classic membrane peeling techniques may be inadequate due to these features, and loosening retinotomy and retinectomy may be required if bimanual techniques and scleral buckling fail to loosen the traction. As a result of advances in surgical techniques, anatomical success rates of 60–80% have been reported in patients with PVR, while functional success rates remain at 30–40% [13–16]. The anatomical success rate in the present study was 67.7%. Although anatomical success rates as high as 90% have been reported with conventional detachment surgery in cases complicated with early stage PVR, anatomical success rates in advanced stage PVR are at the 35–47% level [15, 16]. Hocaoğlu et al. reported that appropriately-timed inferior retinotomy/retinectomy and vitrectomy alone with silicone oil administration for primary or recurrent retinal detachment complicated with anterior inferior PVR resulted in a high primary anatomical success rate (87%) and a good visual acuity level (⩾20/200) in a significant percentage of eyes (80%) [16]. Shalabay et al. reported best corrected visual acuity (BCVA) ≥ 4/60 in 23 eyes, no change in seven (18.5%), and worsening in eight (21%) at six-month follow-up following retinectomy in patients with severe PVR [17]. They also reported no postoperative BCVA better than 6/36 in any patient. Grigoropoulos et al. reported improved visual acuity in 138 eyes (45%), no change in 73 (24%), and worsening in 89 (29%) in 304 patients who underwent retinectomy [18]. When our patients’ preoperative and final control visual acuities were compared, 30(45.45%) patients exhibited improved visual acuity, 10 (15.15%) patients exhibited decreased visual acuity, and no change was determined in 26 (39.39%) patients.

Silva et al. concluded that the etiology of primary retinal detachment impacted significantly on functional outcomes [15]. They reported poorer visual outcomes in cases of rhegmatogenous retinal detachment and traumatic and uveitic retinal detachments, while visual prognosis was better in primary tractional retinal detachment (proliferative diabetic retinopathy and sickle cell anemia) [15]. No statistically significant difference was observed between the rhegmatogenous, tractional and traumatic detachment groups in terms postoperative visual acuity in our study. No significant difference between preoperative and postoperative BCVA was also observed in the rhegmatogenous retinal detachment group, although preoperative and postoperative values differed significantly in the diabetic tractional group and in the traumatic group groups. In our study, BCVA improved in the postoperative period in the diabetic tractional and traumatic groups compared to preoperative values. In our study, we found that the most important variable affecting final visual acuity was the visual acuity present at baseline. In cases with poor baseline visual acuity, the probability of poor vision in the first year increased 6.8 times. We also found that each increase in age reduced the probability of final poor visual acuity by 6.3%.

Garnier et al. reported that retinotomy is indicated in eyes with at least one failed vitrectomy in the majority of cases [19]. The Silicone Study Group reported that 19% of eyes without previous vitrectomy underwent > 180 degree vitrectomy, and Faude et al. reported that 15% of eyes underwent primary 360 degree retinectomy [20, 21]. In the present study, 30.3% of the patients had no previous history of vitrectomy, while 70% had undergone the procedure at least once. Similarly, Hocaoğlu et al. observed no significant relationship between anatomical success and retinotomy/retinectomy width [22]. Those authors reported that the timing of retinectomy may affect success rates and contribute to favorable anatomical results. Hocaoğlu et al. recommended earlier relaxing retinectomy in the development of anterior stage C PVR [22]. Similarly, Silva et al. reported that advanced PVR stages are associated with poor functional outcomes and are more important than PVR localization and width [15]. Silva et al. reported that early PVR stage was associated with good visual prognosis and a 15% increase in the risk of blindness for each grade of PVR [15]. In the current study, no significant difference was observed in recurrence rates in terms of retinectomy width. When we evaluated the recurrence effect of the variables, we found that only age was nearly statistically significant. With each year of age increasing, the recurrence rate decreased by 3.1%. We did not find a significant effect in the other parameters. However, it should also be considered that these results may be due to the low number of cases.

A number of studies have reported an association between the application of prophylactic 360-degree laser retinopexy and a significantly lower rate of retinal recurrent detachment following silicone oil removal (42% vs. 74%) [23, 24]. In the present study, 360 degree laser retinopexy was performed in all cases. Another surgical factor capable of affecting the likelihood of success is the type of tamponade employed. A randomized clinical trial determined no significant difference in reconnection rates between silicone oil and gas tamponade in eyes with severe PVR [25]. In their study, Dimitrios et al. reported an final anatomical success rate of 85% in the gas-treated group and 91.3% in the silicone oil-treated group in patients who underwent retinectomy due to PVR (*p* = 0,46) [26]. However, other retrospective studies have concluded that patients with silicone oil tamponade had significantly better reconnection rates than those with gas tamponade for rhegmatogenous detachment repaired by retinectomy [13]. Tseng et al. reported that the use of silicone oil in eyes undergoing retinectomy for the treatment of severe PVR may improve the initial reconnection rate compared to the use of gas tamponade [14]. Silicone oil was employed as the tamponade in all cases in the present research.

Silva et al. reported a recurrence rate of 23% after silicone oil removal [15]. In this study, recurrence was observed in a total of 22 patients (33%), 12 of which occurred under silicone oil and 10 after silicone oil removal. The use of silicone oil as a tamponade may reduce postoperative dependence on posture, restrict the extent of retinal detachment in the short term, and lower the long-term risk of hypotony. The silicone oil can be left in situ in eyes at high risk for retinal detachment recurrence after silicone oil removal. We determined no difference in recurrence rates according to etiology. Similarly, Beuste et al. reported that the majority of recurrences were caused by proliferative vitreoretinopathy and that anatomical outcomes were independent of the type of PVR (rhegmatogenous, tractional, etc.) [27].

## Study limitations

The principal limitations of this study are its retrospective design, lack of comparative analysis, and small number of cases. In addition, the follow-up periods differed, the etiologies of proliferative vitreoretinopathy differed, and some cases underwent primary retinectomy while others were recurrent cases.

## Conclusion

Retinectomy is a useful approach to the treatment of complicated retinal detachment. Patients who underwent extensive retinectomy exhibited a higher degree of PVR grading. No statistically significant difference in recurrence rates was observed in terms of either etiology or retinectomy size. Furthermore, the better the initial visual acuity, the better the final visual acuity will be.

## Data Availability

The datasets used and analysed during the current study are available from the corresponding author on reasonable request. The data are not publicly available due to privacy or ethical restrictions.
